# Segregating the Effects of Seed Traits and Common Ancestry of Hardwood Trees on Eastern Gray Squirrel Foraging Decisions

**DOI:** 10.1371/journal.pone.0130942

**Published:** 2015-06-25

**Authors:** Mekala Sundaram, Janna R. Willoughby, Nathanael I. Lichti, Michael A. Steele, Robert K. Swihart

**Affiliations:** 1 Department of Forestry and Natural Resources, Purdue University, West Lafayette, Indiana, United States of America; 2 Department of Biology, Wilkes University, Wilkes-Barre, Pennsylvania, United States of America; USDA-ARS, UNITED STATES

## Abstract

The evolution of specific seed traits in scatter-hoarded tree species often has been attributed to granivore foraging behavior. However, the degree to which foraging investments and seed traits correlate with phylogenetic relationships among trees remains unexplored. We presented seeds of 23 different hardwood tree species (families Betulaceae, Fagaceae, Juglandaceae) to eastern gray squirrels (*Sciurus carolinensis*), and measured the time and distance travelled by squirrels that consumed or cached each seed. We estimated 11 physical and chemical seed traits for each species, and the phylogenetic relationships between the 23 hardwood trees. Variance partitioning revealed that considerable variation in foraging investment was attributable to seed traits alone (27–73%), and combined effects of seed traits and phylogeny of hardwood trees (5–55%). A phylogenetic PCA (pPCA) on seed traits and tree phylogeny resulted in 2 “global” axes of traits that were phylogenetically autocorrelated at the family and genus level and a third “local” axis in which traits were not phylogenetically autocorrelated. Collectively, these axes explained 30–76% of the variation in squirrel foraging investments. The first global pPCA axis, which produced large scores for seed species with thin shells, low lipid and high carbohydrate content, was negatively related to time to consume and cache seeds and travel distance to cache. The second global pPCA axis, which produced large scores for seeds with high protein, low tannin and low dormancy levels, was an important predictor of consumption time only. The local pPCA axis primarily reflected kernel mass. Although it explained only 12% of the variation in trait space and was not autocorrelated among phylogenetic clades, the local axis was related to all four squirrel foraging investments. Squirrel foraging behaviors are influenced by a combination of phylogenetically conserved and more evolutionarily labile seed traits that is consistent with a weak or more diffuse coevolutionary relationship between rodents and hardwood trees rather than a direct coevolutionary relationship.

## Introduction

Scatter-hoarding rodents influence seedling establishment in many communities by acting as seed dispersal agents and seed predators [1,2]. Whereas most seeds harvested by rodents are ultimately consumed [1,3], many rodents enhance the probability of germination and establishment by caching in suitable microsites [4] and then subsequently failing to recover a portion of these seeds [1,5]. Most rodents can therefore, easily shift from mutualism to seed predation, and this conditional nature of mutualism between seed-bearing trees and scatter-hoarders is a delicate one [1] that often follows from the rodents’ responses to seed characteristics [2,6].

From the perspective of a rodent, seed preference and seed handling involve a sequence of behavioral decisions. At each step in the process, scatter-hoarders presumably evaluate costs and benefits associated with alternatives to maximize use of resources [7,8]. Experiments with artificial and natural seeds suggest that seed dispersal and handling behavior is related to the perceived value of a seed, or the benefits of attractive traits discounted by the costs levied by defensive seed traits [8,9]. While numerous studies have focused on the relationship between seed traits and fate, fewer have examined the influence of seed traits on the specific behavioral decisions of the scatter hoarder [10,11]. Fine-scale assessments of the seed handling process provide evidence that rodents evaluate the condition of seeds (e.g. by paw manipulation in *Sciurus niger*), which may be predicted by traits indicating seed quality or condition [11].

From the perspective of a tree, some seed traits may improve the probability of being cached or consumed, thereby influencing fitness [12]. Creating an attractive seed (e.g., with a high caloric value) in comparison to that of competitors is beneficial because such seeds may be dispersed longer distances [13,14,15] reducing density-dependent mortality from seedling competitors or seed predation [16–18]. On the other hand, a seed with defensive traits such as a hard shell may be beneficial because such seeds increase handling costs and induce seed caching behaviors thereby increasing seed survival [10,19–22]. Seeds of hardwood trees in China, for example, show trade-offs with respect to investment in seed traits that likely influence handling costs and seed dispersal [12].

Observations of seed handling and seed chemistry provide abundant evidence for reciprocal evolutionary effects between rodents and woody plants [23]. In tree squirrels, suites of behavioral adaptations and morphological adaptations have resulted from selective pressures associated with seed morphology and chemistry [23]. For example, gradients of tannin concentrations have been observed in oak kernels, which impart physiological and metabolic costs to rodents and result in portions of kernels being rejected [24–27]. In response, some rodents have adapted to detoxify tannins [28,29], and many cache acorns with high tannin concentrations more frequently, possibly to avoid or delay the costs associated with ingesting tannins [30,31]. In trees, changes in seed chemistry and morphology have evolved in response to predation and dispersal pressures exerted by tree squirrels [23]. For instance, white oak seeds show no dormancy and multiple-seeded acorns possibly to minimize length of exposure to predation and escape mortality from embryo-excision behaviors by eastern gray squirrels [31,32]. Thus far all approaches to rodent-tree coevolution have collected some combination of detailed morphological, physiological, behavioral, and biogeographic data [23]. While these studies suggest that coevolutionary interactions exist between granivorous rodents and hardwood trees, the strength of this interaction is variable [23].

Coevolutionary relationships between plants and seed dispersers have been described across a continuum, ranging from strong, pairwise interactions to weak, diffuse interactions. Pairwise coevolution occurs between specific species and leads to strong selective pressures on traits [33]. Recent observations of acorn (*Quercus* spp.) embryo excision behavior shown by naïve squirrels (Sciuridae spp.; [31,32]) is an example of a behavioral adaptation and suggests a strong and perhaps pairwise coevolutionary relationship between oaks and squirrels [31,34]. In contrast, weak selective pressures due to disparities in evolutionary rates of woody species and animal dispersers, unpredictability of conditions for seed germination, and a plethora of other factors leads to diffuse or weak coevolution [35,36].

To better understand the nature of coevolutionary interactions between hardwood tree seeds and squirrels, we used a combination of phylogenetic and ecological methods to determine how seed caching and seed consumption behaviors of eastern gray squirrels (*Sciurus carolinensis*) varied as a function of seed traits and phylogenetic relationships of 23 hardwood trees species. Specifically, we hypothesized that gray squirrels would invest more time and travel longer distances to consume and cache seeds with strong physical defenses and high nutrient values. In addition, we predicted that conspecific interference would be related to physical defenses of a seed and nutrient value of a seed. We incorporated phylogenetic relationships of hardwood trees into our analyses to evaluate the relative proportion of gray squirrel seed handling behaviors that is influenced by common ancestry of hardwood tree species and to determine which phylogenetically related seed traits influence handling. We hypothesized that if strong coevolutionary relationships occur between gray squirrels and hardwood tree species, most of the variation in gray squirrel behavior would be explained by seed traits with strong phylogenetic signals. However, weaker, more diffuse coevolutionary relationships would be explained by a mix of seed traits with a strong phylogenetic signal and those traits with no phylogenetic signal. Finally, if no coevolutionary relationships occur, phylogenetically conserved traits should explain a small portion of the variation in gray squirrel behavior.

## Materials and Methods

### Seed handling metrics

Free-ranging eastern gray squirrels on the campus of Purdue University were presented with a sequence of seed types in pseudo-random order between 1 October 2011 and 15 February 2012. Seeds were obtained from commercial seed companies (F. W. Schumacher Co., East Sandwich, MA and Sheffield’s seed Co., Locke, NY) and from beneath trees on campus. Seeds were stored at 4°C, separated by species in plastic containers. Seed presentations were conducted by tossing a seed to a squirrel from 1–4 m. If the squirrel retrieved the seed, the observer video recorded the event until the seed was consumed or cached. When the seed was consumed, the observer recovered the remains of the seed kernel and shell by sifting through the litter and soil. The distance a squirrel moved to cache or consume a seed was determined by retracing the path of the squirrel, marking the final destination, and then measuring the straight-line distance between the point of encounter of the seed and the final location. If the distance travelled was too great to be accurately estimated with a tape measure, a GPS (Garmin Model No.72) was used to obtain coordinates. Straight-line distance was then calculated in ArcGIS 10 (Environmental Systems Research Institute 2010). A second seed was presented to a squirrel only after the first seed was handled, and the process repeated until squirrels no longer recovered seeds. Presentations of the same seed types were spatially separated by selecting locations that were >100 m apart to increase the likelihood that data were collected from different squirrels. Exceptions were permitted only if two different squirrels could be recorded simultaneously. The protocol included non-intrusive observation with no hindrance to the animals, and therefore, we did not seek additional approval from Purdue Animal Care and Use Committee (PACUC).

Foraging trial videos were collected for 23 seed types from the tree families Betulaceae, Fagaceae, and Juglandaceae (S1 Table). The time taken to consume or cache each seed was calculated from the videos. Time required to consume and cache a seed was defined as the time needed to consume and completely bury a seed respectively, and did not include travel time. We quantified the number of recorded trials per seed type in which the focal squirrel was chased by a conspecific while handling a seed, which we define as conspecific interference [37].

### Seed traits

The value of each seed trait was measured for 3 seeds for each of the 23 seed types unless specified otherwise (S2 Table). Percentage of moisture in seeds was calculated by drying samples at 103°C for 3 days. Percentage of tannic acid equivalents in nutmeat was determined by performing radial diffusion assays [38,39]. The value was expressed as tannic acid equivalents occurring in 100 grams of dry nutmeat. Energy content of the nutmeat was obtained in a bomb calorimeter (PARR 1262 bomb calorimeter, Parr Instrument Co., Moline, IL) using benzoic acid as a calibration standard. The value was adjusted to obtain calories per gram of dry weight. Seed hardness was estimated as the peak load (kilograms) required to break a seed’s shell and pierce the nutmeat. Testing was done with a MTS/Sintech computerized testing machine (MTS Corporation, Eden Prairie, MN) using a crosshead fashioned after the skull of an eastern gray squirrel and designed to mimic a squirrel’s incisor action (S1 Fig). Only the upper incisors were used to obtain hardness estimates. Peak load within 5 mm crosshead displacement was sufficient to break the seed shell of most seeds and was used as an estimate of seed hardness. Only butternut (*Juglans cinerea*) seed shells were not pierced with the 5 mm crosshead; therefore, 10 mm displacement was employed for this seed. Seed shell thickness was determined by visualizing the cross section of seed shells with a stereoscopic microscope attached to a Nikon Imaging System at 4.91 micrometers per pixel. Proximate analysis was performed following Association of Official Analytical Chemists (AOAC) protocols to estimate percentage of crude proteins (Kjeldahl, AOAC Official Method 984.13 A-D, 2006), fats (ether extraction, AOAC Official Method 920.39 A, 2006) and carbohydrates (difference method, FAO 2003) in nutmeat. We combined kernels from multiple seeds to obtain at least 10g of dried material, and duplicate samples were used in proximate analyses for each seed type. Finally, we used number of days of cold stratification required before germination in a chamber as an index of dormancy period of seed types. Estimates of cold stratification days were obtained from the literature [40–42], and averages were computed and used whenever a range of days was provided.

### Molecular and phylogenetic methods

We conducted a phylogenetic analysis to estimate and incorporate evolutionary relationships between hardwood tree species into analyses in this study. To achieve this goal, we first extracted DNA from plant tissue using two methods. For the majority of species, we extracted DNA from leaf tissue using a phenol-chloroform protocol [43], modified by grinding fresh leaf tissue with 500mg PVPP in liquid nitrogen [44]. We used the PowerPlant Pro DNA Isolation Kit (MoBio Laboratories, Inc; Carlsbad, CA) to extract DNA from *Carya tomentosa* leaf tissue and embryos removed from the seeds of Quercus prinus and Quercus bicolor.

To create a phylogeny for our 23 hardwood tree species, we used sequence data from two chloroplast genes- ribulose-bisphosphate carboxylase oxygenase large subunit (rbcL) and maturase K (matK) as well a portion of the nuclear genome that included the internal transcribed spacer 1, 5.8S rRNA gene, and the internal transcribed spacer 2 (ITS). When possible, we obtained relevant sequences from Genbank (S6 Table). For the remaining species, we sequenced each sample using polymerase chain reaction (PCR) on an Eppendorf Mastercycler (Eppendorf, Westbury, New York). Initial amplification was performed in 20 μL reactions containing approximately 40 ng template DNA, 1 unit of NEB Taq polymerase, 0.3 lM of each primer, 1.5 mM MgCl2, 10 mM Tris-HCl, 50 mM KCl, 0.5 mg/ml BSA, and 0.2 mM of each dNTP. PCR parameters included an initial denaturing temperature of 95°C for 30 seconds followed by 30 cycles of 95°C for 30 seconds denaturing, annealing temperature of 53°C for rbcL and matK [45] and 58°C for the ITS gene (primers ITS5 and ITS4; [46]), and 72°C for 1 minute elongation, with a final elongation temperature of 72°C for 5 minutes. We cleaned the resulting PCR product with the MinElute PCR Purification Kit (Qiagen, Valencia, California). Sanger sequencing reactions were performed at 10μL and contained 50 ng of template and Big Dye 3.1. We used an ABI Prism 3730XL sequencer (Applied Biosystems, Foster City, CA, USA) and trimmed sequences in Sequencher 4.7 (Gene Codes Corp., Ann Arbor, MI).

We aligned the sequences for each gene using M-coffee web server hosted by the Centre for Genomic Regulation [47,48]. We tested for congruence of species pairwise distance matrices derived for the 3 aligned genes in this study by calculating Kendall’s W statistic of concordance as implemented by the function ‘CADM’ within the package ‘ape’ in R [49]. After rejecting the null hypothesis of incongruence between distance matrices, we concatenated sequences of the 3 genes for further analyses. The function ‘modelTest’ in the package ‘phangorn’ was used to select the best nucleotide substitution model for the concatenated sequences [50]. Model SYM (symmetrical model; [51]) + G (gamma distributed rate variation) was selected based on BIC values.

We generated a phylogeny for our 23 species using the program BEAST [52], and prepared our input file using BEAuti [53]. We used a relaxed lognormal molecular clock [54], the Yule speciation model [55,56], and *Rubus occidentalis* as an outgroup. We ran 5 independent runs of 50 million steps, thinning to every 1000 trees. We confirmed convergence of each run with Tracer, and filtered the first 10% each group of runs using TreeAnnotator.

### Statistical analyses

We estimated pairwise Spearman rank-correlations between the seed handling metrics and all seed-trait values in this study. We also computed Moran’s I metric of phylogenetic signal [57] for all seed traits and evaluated significance by permuting seed trait values across tips of the phylogeny 1000 times. For all further statistical analyses, we included the following seed traits in our seed trait matrix: percent lipids, percent carbohydrates, percent proteins, caloric concentration, dormancy period, log hardness, log shell thickness, log interaction of hardness and thickness, log kernel mass, log shell mass, and log tannin concentration. Natural log transformation was used for seed traits with skewed distributions. Before log-transforming tannin concentrations, a low value of 0.35% TAE was assigned to the 3 seeds with 0% TAE to avoid undefined numbers.

We incorporated the phylogeny of hardwood trees into our statistical analyses using two methods—variance partitioning analyses and phylogenetic PCA. To partition the variation in each seed handling metric between seed traits and phylogenetic relationships, we used phylogenetic eigenvectors and a partial regression analysis implemented in the R package ‘PVR’ [58]. The PVR method converts a matrix of double-centered phylogenetic distances into eigenvectors, which are then used as predictors to explain variation in a trait of interest. The first few eigenvectors, which capture most of the variation in the distance matrix, represent differences between clades at the root of the phylogeny. Subsequent eigenvectors capture variation among taxa or groups closer to the tips. Selection of all eigenvectors as predictors in regressions is not necessary and may result in large or inflated R^2^ values. In contrast, selection of too few eigenvectors can result in residual autocorrelation because all of the phylogenetic dependence is not captured. For our analyses, we selected phylogenetic eigenvectors that minimize residual autocorrelation as estimated by Moran’s I [59,60]. The PVR method then uses the phylogenetic eigenvectors and environmental variables (i.e. seed traits) as predictors in a partial regression analyses to partition the variation in a trait. We used the PVR method to obtain the variation in each seed handling metric (handling time and distances travelled prior to handling for cached and consumed seeds) explained by seed traits alone, selected phylogenetic eigenvectors alone, information shared between seed traits and selected phylogenetic eigenvectors, and finally unexplained factors.

Next, we identified linear combinations of seed traits that exhibit autocorrelations with the hardwood tree clades observed in our phylogeny. We performed a phylogenetic principal components analysis (pPCA) using the Abouheif method (equivalent to Moran’s I metric) of computing phylogenetic signals [61]. The pPCA groups seed traits into possible ‘global’ and ‘local’ structures given a candidate phylogeny and traits of interest. The ‘global’ structures constitute pPC axes that are positively correlated to clades at the root of the phylogeny. Large positive eigenvalues represent axes showing large variance and positive phylogenetic autocorrelation or Moran’s I. In contrast, ‘local’ structures constitute pPC axes and traits with negative phylogenetic autocorrelation indicative of traits that are different between related taxa. Large negative eigenvalues represent axes explaining a large variance and axes with a negative Moran’s I. Together, the global and local pPC axes can suggest specific life history strategies adopted by the taxa. We performed a pPCA on our seed trait matrix using the function ‘ppca’ in the package ‘adephylo’ [62]. To ensure that inconsistencies in the phylogeny were not driving the observed relationships, we collapsed subclades into groups and performed the pPCA a second time. The collapsed clades in our phylogeny included-walnuts (*Juglans*), hickories (*Carya*), white oak group (*Quercus* section Quercus), red oak group (*Quercus* section Lobatae), and chestnuts (*Castanea*). In addition, the tree contained the following singleton taxa—hazelnut (*Corylus americana*), beechnut (*Fagus grandifolia*) and tanoak (*Notholithocarpus densiflorus*). We retained the first three pPC axes (2 global and 1 local as determined by the sign of the eigenvalue) explaining the highest proportion of variation. We tested our selected pPC axes for significant positive and negative phylogenetic autocorrelation by performing Abouheif’s test on the PC scores using the ‘abouheif.moran’ function. Finally, we regressed the 4 squirrel seed handling metrics in 4 different multiple linear regression models against our 2 global and 1 local pPC axes. Plots of residuals indicated unequal variances and departures from normality, so we evaluated significance of predictors by permuting the response variable 1000 times and estimating the null distribution of coefficients. Finally, we performed a post-hoc Poisson regression to predict number of trials where a conspecific interfered with seed handling using our 3 pPC axes as predictors. To evaluate if interference caused an increase in handling time, we performed a paired t-test comparing average times spent handling a seed type when interference was not observed to when interference was not observed pooling across seed types. All analyses were performed in R 3.1.0.

## Results

We recorded 272 foraging trials, which included 5–7 seed-consumption and 4–6 seed-caching trials per seed type (mean of 5.04 seed-consumption and 5.52 seed-caching trials/seed type). For tanoak (*N. densiflorus*) 4 caching trials were recorded. For black walnut (J. nigra), the distance travelled to consume a seed was computed for 4 of the 5 consumption trials. Kernels were typically consumed or cached (S1 Table).

Pairwise Spearman rank correlations between seed traits of the 23 seed types revealed several patterns of correlation between groups of seed traits (S3 Table). Nutrient and perishability-related variables such as percentage of proteins, carbohydrates and lipids in the kernel, caloric concentration, and dormancy period were positively correlated. Physical defensive traits including hardness, shell thickness, hardness and thickness interaction, and dry mass of shell were also positively correlated. In addition, lipid concentration was positively correlated to shell thickness and hardness. Tannin concentration of kernels was correlated to protein concentration and dormancy period only (S3 Table).

**Fig 1:**
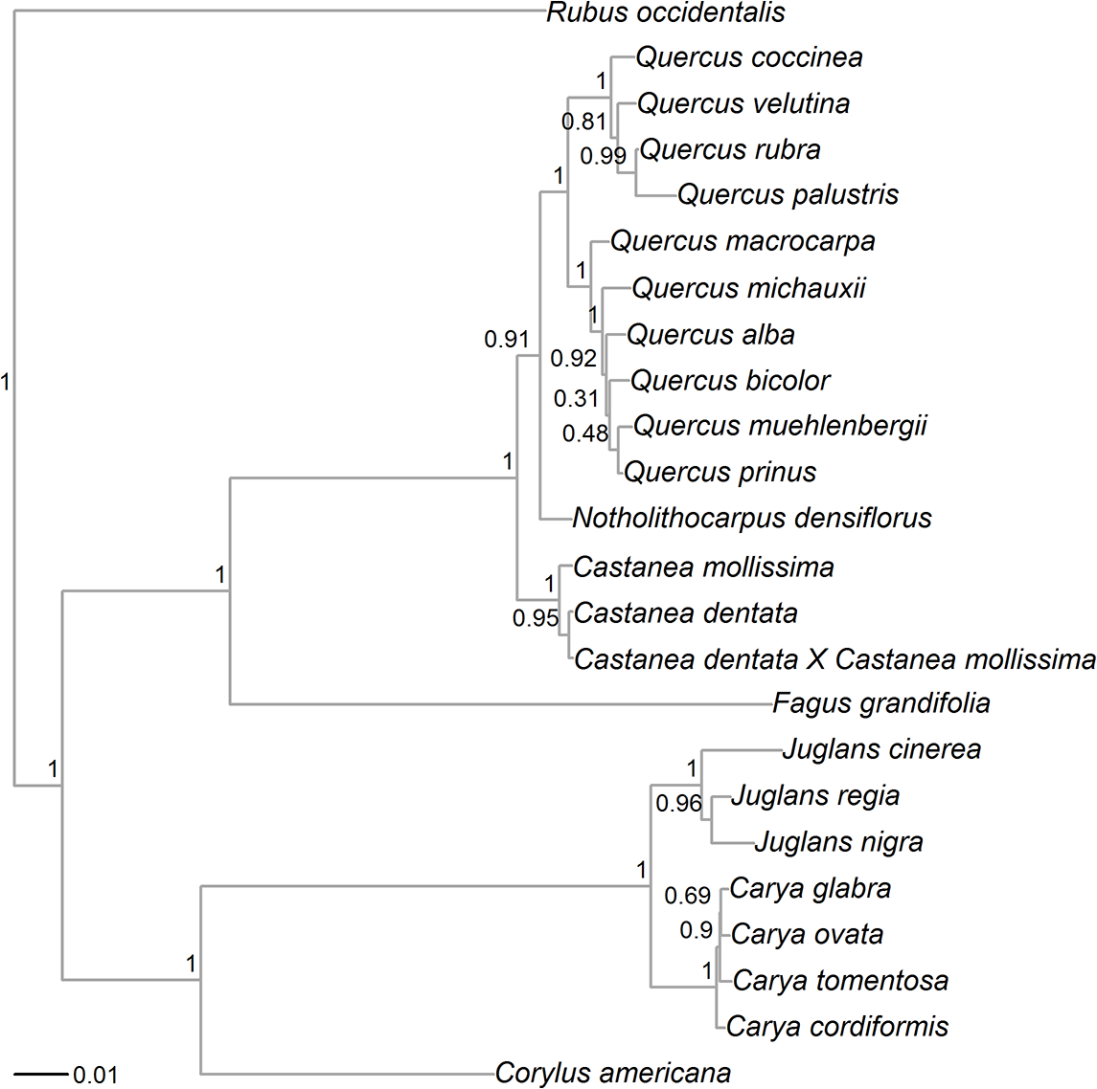
Bayesian maximum clade credibility tree for 23 hardwood tree species from the families Fagaceae, Juglandaceae and Betulaceae, using *Rubus occidentalis* as an outgroup. Tree inferred from rbcL, matK and ITS gene sequences. Posterior node support indicated by node labels.

We successfully extracted DNA from plant tissue for 12 taxa for which sequences were not available in GenBank (S6 Table). The concatenated dataset contained 1864 sites, out of which 332 were parsimony informative. Topology of the Bayesian tree (Fig 1) is supported by other existing phylogenies and current taxonomic classification of the hardwood trees species [63–67]. In addition, posterior probabilities associated with the clades were usually >0.9. Weak node support occurred only at the tips of the phylogeny within the white oak group (*Quercus* section Quercus), and the split of shagbark and pignut hickory (*Carya glabra* and *Carya ovata*, Fig 1).

After deconstructing our phylogeny into eigenvectors, we performed variance partitioning to estimate the proportion of squirrel seed handling investments explained by seed traits and phylogeny (Table 1). Seed trait variation alone explained between 27 and 73% of the variation in the seed handling behavior of squirrels. Phylogeny indepdent of the measured traits explained <6% of all seed handling behaviors. For time required to consume a seed and distance travelled to cache a seed, the variation explained by the combination of seed traits with a phylogenetic structure was 44% and 56%, respectively. Unexplained variation was relatively high (21% and 32%) for distance moved to consume a seed and time required to cache a seed.

**Table 1:** Variance partitioning results for each squirrel seed handling behavior, including time to consume, distance travelled to consume, time to cache, and distance travelled to cache a seed.

Response variable	Seed traits alone	Combined traits & phylogeny	Phylogeny alone	Unexplained
Time to consume	0.502	0.440	0.006	0.052
Distance travelled to consume	0.731	0.055	0.000	0.214
Time to cache	0.409	0.213	0.058	0.320
Distance travelled to cache	0.274	0.556	0.004	0.156

Values are proportion of variation (R^2^) of each behavior explained by seed trait information alone, combined information between seed trait and phylogeny, phylogenetic information alone, and unexplained sources of variation.

**Fig 2:**
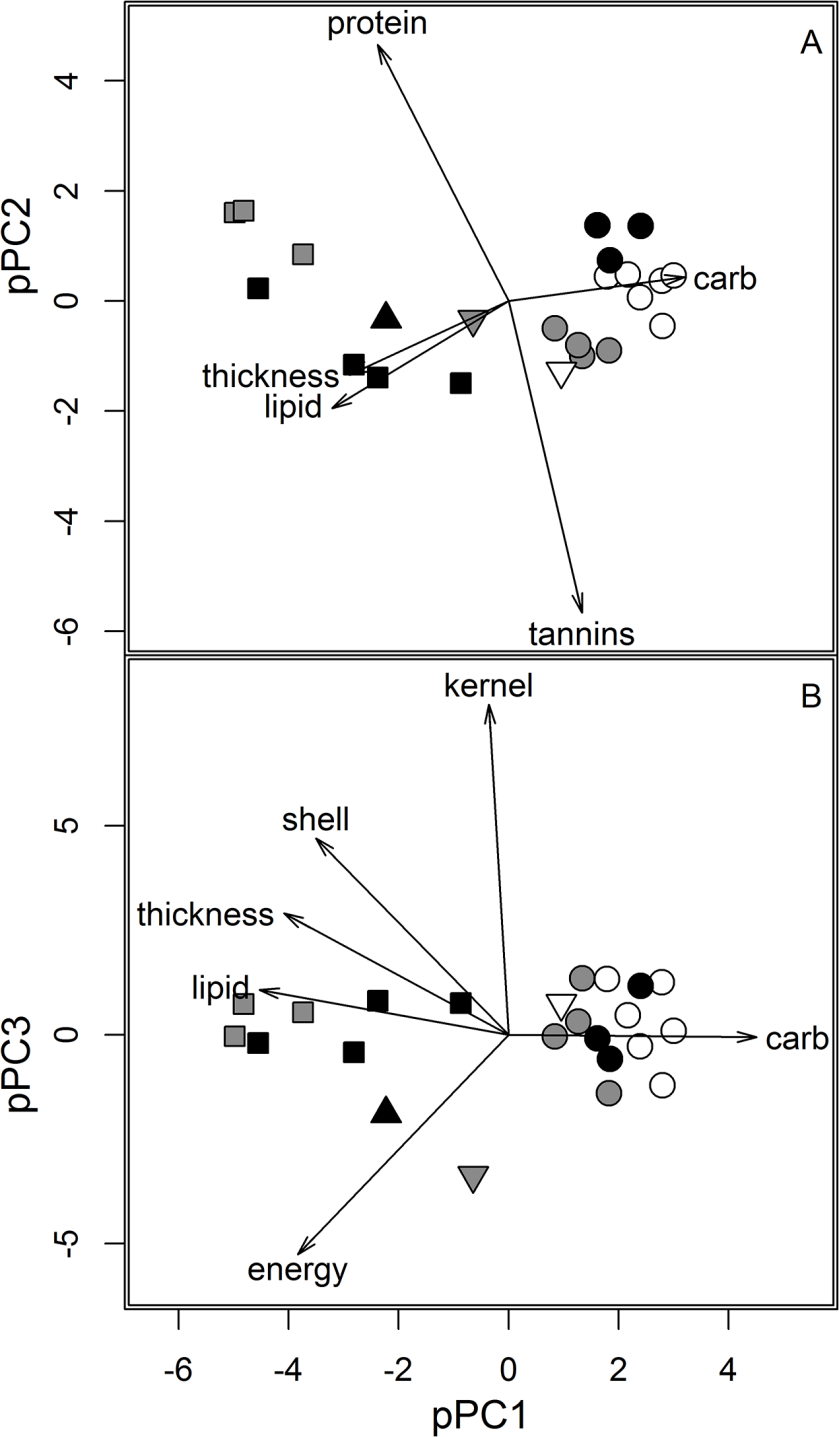
Biplots of seed traits and scores of 23 hardwood tree species obtained from a phylogenetic principal components analysis (pPCA). Distribution of hardwood tree species across the first 2 ‘global’ pPC axes (A). Distribution of tree species across the first ‘global’ and third ‘local’ pPC axes (B). Separation of species belonging to families Juglandaceae and Betulaceae (symbols: square and upright triangle) from Fagaceae (symbols: circles and inverted triangle) is observed across phylogenetically autocorrelated pPC1. Separation of *Juglans* (gray filled square) from *Carya* (black filled square) and separation of *Castanea* (black filled circle) from *Quercus* section Quercus (open circle) and *Quercus* section Lobatae (gray filled circle) is seen across pPC2. The third axis is not significantly phylogenetically autocorrelated and species are not differentiated by taxonomic clades across pPC3. Biplots also include *Notholithocarpus densiflorus* (inverted gray triangle) and *Fagus grandifolia* (inverted open triangle). Biplot arrows plotted only for seed traits with loadings greater than 75^th^ percentile of absolute loadings (pPC loadings and 75^th^ percentile cutoff in S4 Table).

The 11 seed attributes of 23 seed types (Fig 2, S4 Table) were reduced to three phylogenetic principal components (pPC), cumulatively explaining 83.3% of the total variation. We interpreted the first global pPC axis as family-level differences between seeds. This axis accounted for 63.5% of the total variation. Percentage of carbohydrates loaded highly and contrasted with percentage of lipids and shell thickness. Scores from pPC1 were phylogenetically autocorrelated (I = 0.83, p = 0.002), and differentiated lipid-rich, thick shell seeds of Juglandaceae and Betulaceae from carbohydrate-rich Fagaceae (Fig 2). The second global pPC axis explained 8.4% of the variation, and reflected genus and section-level differences in seeds. The axis positively loaded protein concentration, and negatively loaded tannin concentration and dormancy period. Scores from pPC2 axis were also phylogenetically autocorrelated (I = 0.36, p = 0.004) and separated high protein *Juglans* seeds from *Carya* seeds and high protein *Castanea* seeds from low dormancy *Quercus* section Quercus seeds (Fig 2). Finally, the third pPC axis reflected kernel size of seeds and explained 12.4% of the total variation. This axis positively loaded kernel and shell mass and negatively loaded energy value of seed. Scores from pPC3 were not significantly phylogenetically autocorrelated (I = -0.12, p = 0.694, Fig 2). The pPC axes did not change substantially after reducing the phylogeny to only well-established clades.

Time required to consume a seed was predicted by the two global pPC axes and the local pPC axis (R^2^ = 0.76, Table 2). The distance moved to consume a seed was positively predicted predominantly by the local pPC axis and marginally by the first global pPC axis (R^2^ = 0.30, Table 2). Time required to cache a seed was negatively predicted by the first global pPC axis and positively to the local pPC axis (R^2^ = 0.43, Table 2). Distance moved to cache a seed was correlated negatively with the first global pPC axis and positively with the local pPC axis (R^2^ = 0.76, Table 2).

**Table 2:** Regressions of squirrel foraging behavior (time to consume, distance travelled to consume, time to cache, and distance travelled to cache a seed) against 3 phylogenetic PC axes (pPC1, pPC2, pPC3).

Response variable	Predictor	Slope	t-statistic	p-value
Time to consume a seed	Intercept	9.216	7.039	**0.000**
	pPC1	-3.112	-6.208	**0.000**
	pPC2	4.196	3.039	**0.006**
	pPC3	2.843	2.388	**0.033**
Distance moved to consume a seed	Intercept	6.048	5.341	0.072
	pPC1	-0.736	-1.699	0.090
	pPC2	-0.007	-0.006	0.994
	pPC3	2.460	2.389	**0.039**
Time to cache a seed	Intercept	2.090	10.044	**0.039**
	pPC1	-0.220	-2.864	**0.016**
	pPC2	0.130	0.614	0.544
	pPC3	0.507	2.133	**0.047**
Distance moved to cache a seed	Intercept	26.283	12.400	**0.000**
	pPC1	-5.263	-6.757	**0.000**
	pPC2	0.437	0.204	0.840
	pPC3	8.742	3.621	**0.002**

Estimates of slope, t-statistic and p-value are provided for each predictor. Boldface depicts values of p < 0.05; italics depict 0.05 ≤ p < 0.10.

Competitive interference was observed in 10 seed types (range 0–3 trials per seed type) and was observed in 3 seed-caching videos and 11 seed-consumption videos. Number of trials in which conspecific interference was observed was not predicted by any of the pPC axes. The mean handling (consumption and/or caching) time did not differ within a seed type between trials in which interference was observed and trials where interference was not observed (Paired t-test t = 0.22, df = 10, P = 0.95).

## Discussion

Our results provide evidence that foraging investments are influenced by a mixture of seed traits that are phylogenetically autocorrelated (mass of shell, hardness of shell, shell thickness, lipid concentrations, and carbohydrate concentrations) and those that are not (kernel size and tannin concentrations, see Table 2, S5 Table). We suggest that these results support the existence of a diffuse coevolutionary relationship between eastern gray squirrels and hardwood tree seeds.

By incorporating phylogenetic information, we found that gray squirrel foraging investments are influenced to different degrees by seed trait information, phylogenetic relatedness of hardwood trees and unexplained sources of variation. Distance moved to consume a seed and time to cache a seed showed a relatively high degree of unexplained variation in the variance partitioning analyses, suggesting that these metrics may be influenced to a relatively greater degree by variables not measured in this study. A different pattern of variance partitioning was observed for time required to consume a seed and distance to cache a seed, both of which are behaviors related to caching decisions and seed survival. Specifically, seeds with large handling times may be cached more often, increasing the possibility of germination and establishment, and seeds cached at long distances may escape density-dependent sources of mortality [16,17,18,68]. These behaviors were explained to a large extent by the shared information in seed traits and phylogeny (Table 1), suggesting possible coevolution between hardwood trees and eastern gray squirrels.

Coevolution results from interactions that occur over long time scales and should extend to ancestral states existing prior to differentiation of genera [36]. Our pPCA methods allowed us to determine which seed trait variants correspond to family and genus-level differentiations among our 23 hardwood tree species. By regressing squirrel foraging investments against pPC axes, we indirectly determined if gray squirrels are sensitive to family or genus-level seed trait variations.

Family-level seed trait differences were more important in predicting gray squirrel behavior than genus- or section-level differences. The earliest split observed in our hardwood tree phylogeny corresponds to the separation of Juglandaceae and Betulaceae from Fagaceae (Fig 1), which coincides with two different strategies of making seeds (as suggested by the global pPC1 axis)—lipid-rich and thick shelled seeds in Juglandaceae and Betulaceae, as opposed to thin-shelled but carbohydrate-rich Fagaceae seeds (Fig 2). Specifically, squirrels travel farther to cache and invest more time caching/consuming lipid-rich and thick-shelled Juglandaceae and Betulaceae seeds (Table 2), potentially improving seed survival and fitness of these trees. The second pPC axis, describing differences in seed traits between genera and section clades, was also included as a predictor in our regression analyses but positively explained only time to consume a seed (Table 2). This finding supports the existence of coevolutionary interactions between squirrels and hardwood trees.

If coevolutionary interactions exist between gray squirrels and hardwood trees, either a weak diffuse coevolution or a strong pairwise coevolution could exist. The local axis, with no phylogenetic signal, was an important predictor of squirrel behavior and thus supports a diffuse coevolutionary interaction. Specifically, large loadings on the local pPC3 axis reflected high kernel mass, which was one of two seed traits in our set showing no phylogenetic signal (S4 and S5 Tables). This local axis explained only 12% of seed trait variation, but it was a significant predictor of distance travelled to consume or cache a seed and time to cache a seed. If kernel size is an evolutionarily more labile trait, then trees from all the three families can improve chances of recruitment by producing seeds with larger kernels to attract dispersers and increase handling costs.

The adaptive strategies of trees to influence seed handling are not limited to attributes of a single seed. Trees with smaller kernels and smaller seed mass may adopt strategies not examined in this study such as production of large numbers of seeds and synchronized masting [69,70]. Environmental variables not considered here could also influence foraging behaviors, particularly behaviors with a relatively high proportion of unexplained variation including number of conspecific interferences per seed type, time to cache a seed and distance to consume a seed. These foraging behaviors may be influenced by environmental metrics such as density of conspecifics at a location and perceived predation risk. However, seed traits alone accounted for 73% of distance travelled to consume a seed and 41% of time to cache a seed in variance partitioning analyses, which were larger than the R^2^ values associated with the multiple regression analyses involving 2 global and 1 local pPC axes. Thus, additional local pPC axes showing no significant phylogenetic signal, and not considered here, might play an important role in explaining seed handling behaviors.

Although the small sample (n = 3) of seeds could lead to imprecise estimates for attributes, our seed trait values match published values [68], which also indicate that seeds of Juglandaceae have higher energetic values and lipid content as opposed to seeds of Fagaceae. Given the large differences in seed traits among species, trait variation within species is unlikely to substantially alter the phylogenetic signals computed here. That said, the effects of seed traits on the fitness of individual trees will depend on their specific environmental and competitive contexts. Therefore, future studies that directly address intraspecific trait variation will be needed to completely understand the coevolutionary dynamics of trees and rodents.

From a gray squirrel’s perspective, the degree to which seed traits influenced foraging investments differed depending on whether the seed was consumed or cached. For consumed seeds, degree of physical protection, caloric potential of seed and net protein availability influenced handling behaviors (Table 2). Consistent with other studies [20], consumption time increased for large seeds with thick, hard shells. Consumption time was also negatively related to tannin concentration of the kernel, which may result from squirrels rejecting portions of kernels with high tannins (~0.25–0.5g), potentially because high tannin concentrations render a seed unpalatable [24]. Distances to consume and cache seeds were influenced by lipid concentration, shell thickness, and kernel size, which is consistent with other studies [13,71]. Moreover, we observed a significant positive correlation between handling time and distance moved to consume a seed (r = 0.674, S3 Table) which suggests that squirrels may carry seeds farther to sites where predation risk or conspecific competition is low before consuming seeds that require more time and energy to handle [10].

We found no evidence of interference from conspecifics varying with respect to seed traits. Paired t-test results suggest that conspecific interference does not influence time required to consume a seed. However, we evaluated interference post-hoc, and our results were based on only 14 instances. Therefore, studies designed to examine effects of interference from conspecifics are needed to confirm our results. For seeds that were cached, we found increased cache time for seeds with a thick shell and high lipid concentration (pPC1), and large kernel size (pPC3). These results indicate that large seeds require a deeper site to be excavated for caching. Alternatively, this result could also arise if squirrels perceive large seeds to be valuable and consequently invest longer amounts of time in caching them [72].

Our results reinforce the notion that different seed traits can influence foraging investments related to caching versus consumption. Moreover, our results suggest that it is unwise to discount the behavioral importance of seed traits that show no phylogenetic structure or explain little variation in trait-phylogeny space. Future research using phylogenetic methods in concert with trait measurements and behavioral observations will be useful to compare the effects of selection pressures imposed by rodents on seed-bearing trees in ecosystems that presumably vary in their coevolutionary histories.

## Supporting Information

S1 Fig Cross-head design based on the skull of an eastern gray squirrel (*Sciurus carolinensis*) used with an MTS/Sintech machine to estimate hardness of the 23 hardwood trees in this study. Upper and lower jaws were separated by removing the screw holding the two pieces together.
(JPG)

S1 Table Eastern gray squirrel (*Sciurus carolinensis*) foraging behaviors associated with 23 seed hardwood tree seeds. Metrics include average time to consume (mins), average distance travelled to consume (m), average time to cache (mins), average distance travelled to cache (m) and average grams of unconsumed kernel per seed type (g).
(PDF)

S2 Table Seed trait values for the 23 hardwood tree species in this study. Traits include total seed mass (g), shell mass (g), hardness (kg), shell thickness (micrometers), cold stratification days required to break dormancy (d), energetic or caloric value (calories per gram), protein (%), carbohydrate (%) and lipid (%) content, tannin concentration (% tannic acid equivalents), and moisture content (%). * Seeds of genus *Castanea*, $ Seeds of genus *Corylus*, ¥ Seeds of genus *Carya*
(PDF)

S3 Table Pairwise Spearman rank correlation tests for squirrel seed handling metrics and seed traits. Spearman rank correlation coefficients are in the upper diagonal of matrix and accompanying p-values in the lower diagonal. Boldface values correspond to significant correlations at an alpha of 0.05. In the table, kern = kernel mass (g), shell = shell mass (g), tannins = percentage TAE, energy = calories/g, hard = hardness (kg), thick = thickness (micrometers), interaction = hardness * thickness, dormancy = average cold stratification days, protein = percentage proteins in kernel, carb = percentage carbohydrate in kernel, lipid = percentage lipid in kernel, cons. time = consumption time (mins), cons. dist = distance to consume a seed (m), cache. time = time to cache (mins), dist. cache = distance to cache a seed (m).
(PDF)

S4 Table Phylogenetic principal components (pPCA) loadings and 75^th^ percentile or cutoff of absolute loadings associated with 11 seed traits. Traits include kernel mass (log), shell mass (log), tannin concentration (log), energetic or caloric concentration, hardness (log), shell thickness (log), interaction of hardness and shell thickness (log), dormancy period (given by number of cold stratification days), protein, carbohydrate and lipid concentration for each axis. Boldface values represent loadings that fall outside of 75^th^ percentile cutoff values and are variables that are weighted heavily by the corresponding axis.
(PDF)

S5 Table Moran’s I indicating degree of phylogenetic autocorrelation for each seed trait. * The p-value is given as proportion of null distribution (null distribution estimated by randomizing trait values across tips of the phylogeny) greater than estimated Moran’s I value. Boldface p-values are significant at alpha of 0.05.
(PDF)

S6 Table GenBank accession numbers for sequences used to create the phylogenetic tree. * Sequences generated for this study.
(PDF)
