# Supplementary figures and images for: Segregating the Effects of Seed Traits and Common Ancestry of Hardwood Trees on Eastern Gray Squirrel Foraging Decisions
Source: PLoS One. 2015 Jun 25;10(6):e0130942. doi: 10.1371/journal.pone.0130942 (PMC4482146; doi:10.1371/journal.pone.0130942)

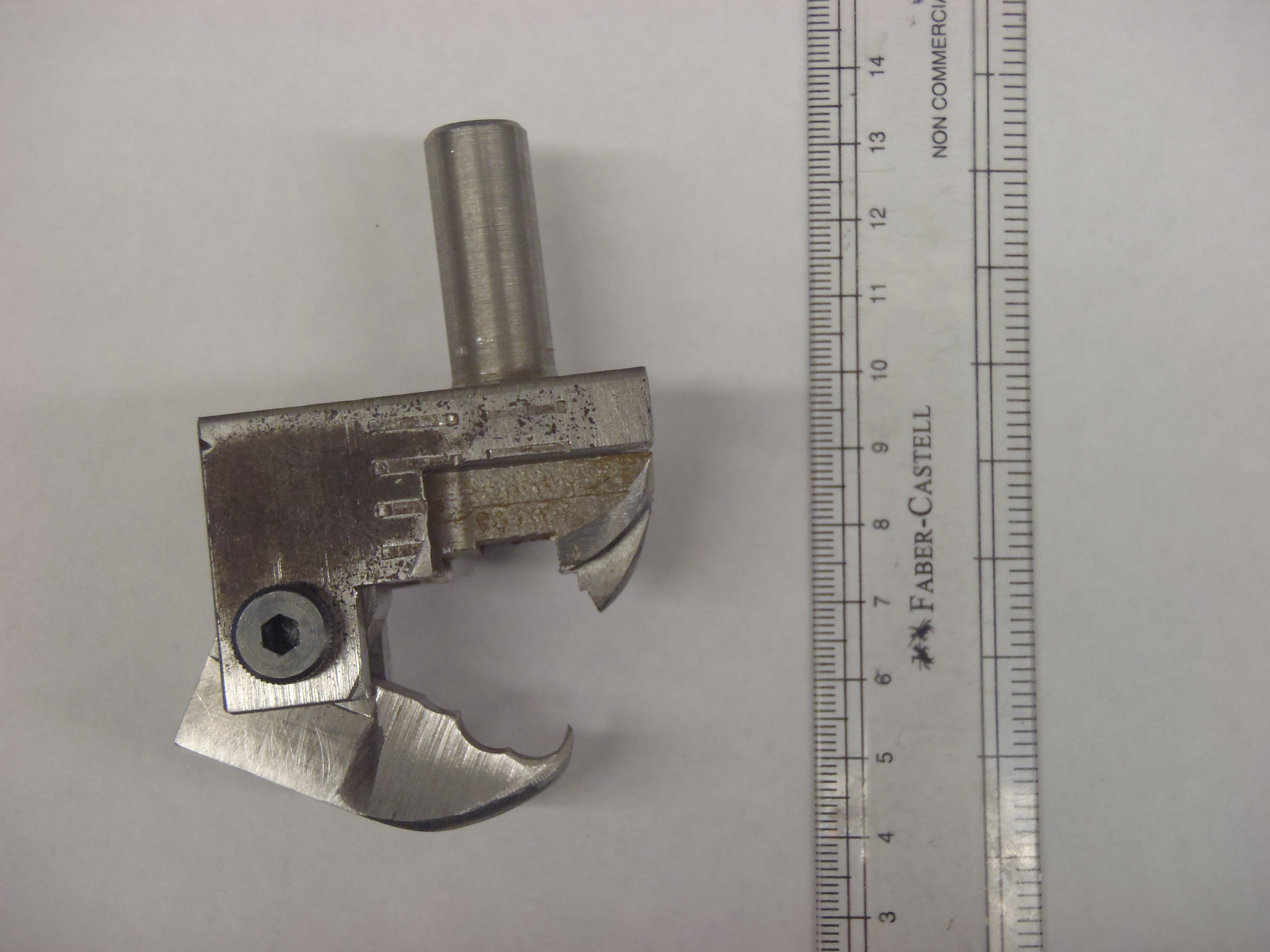

Supplement: S1 Fig — Upper and lower jaws were separated by removing the screw holding the two pieces together. (JPG) [file pone.0130942.s001.JPG]
