# Supplementary material for: Segregating the Effects of Seed Traits and Common Ancestry of Hardwood Trees on Eastern Gray Squirrel Foraging Decisions
Source: PLoS One. 2015 Jun 25;10(6):e0130942. doi: 10.1371/journal.pone.0130942 (PMC4482146; doi:10.1371/journal.pone.0130942)
Supplement: S1 Table — Metrics include average time to consume (mins), average distance travelled to consume (m), average time to cache (mins), average distance travelled to cache (m) and average grams of unconsumed kernel per seed type (g). (PDF) [file pone.0130942.s002.pdf]

Seeds	Time to consume (mins)	Distance to consume (m)	Cache time (mins)	Distance to cache (m)	Unconsumed kernel (g)
Fagus grandifolia	0.3	0	NA	NA	0
Castanea dentata X Castanea mollissima	2.74	7.18	0.77	10.96	0
Castanea dentata	4.16	8.18	0.95	15.43	0
Castanea mollissima	8.33	3.74	3.45	40.34	0
Notholithocarpus densiflorus	3.51	24.17	1.86	35.13	0
Quercus alba	2.42	1.15	1.67	5.66	0
Quercus bicolor	6.55	3.43	1.9	17.7	0
Quercus macrocarpa	2.51	3.11	0.79	2.87	0
Quercus michauxii	8.07	9.21	2	24.01	0
Quercus muehlenbergii	1.24	1.83	0.94	5.58	0
Quercus prinus	6.24	4.32	2.44	13.06	0.47
Quercus coccinea	2.6	2.72	1.06	27.09	0
Quercus palustris	0.84	0.61	0.93	10.98	0
Quercus rubra	5.56	4.57	2.92	25.82	0.29
Quercus velutina	2.15	2.42	3.08	21.16	0
Corylus americana	1.33	0.61	0.69	4.4	0
Carya cordiformis	13.99	3.59	3.69	39.39	0
Carya glabra	13.03	15.78	1.4	38.17	0
Carya ovata	6.88	1.41	2	45.17	0
Carya tomentosa	22.33	2.92	4.33	46.89	0
Juglans cinerea	46.36	11.83	3.75	54.08	1.41
Juglans nigra	38.79	13.87	3.54	59.82	1.8
Juglans regia	12.05	12.46	3.43	60.76	0
