# Supplementary material for: Segregating the Effects of Seed Traits and Common Ancestry of Hardwood Trees on Eastern Gray Squirrel Foraging Decisions
Source: PLoS One. 2015 Jun 25;10(6):e0130942. doi: 10.1371/journal.pone.0130942 (PMC4482146; doi:10.1371/journal.pone.0130942)
Supplement: S2 Table — Traits include total seed mass (g), shell mass (g), hardness (kg), shell thickness (micrometers), cold stratification days required to break dormancy (d), energetic or caloric value (calories per gram), protein (%), carbohydrate (%) and lipid (%) content, tannin concentration (% tannic acid equivalents), and moisture content (%). * Seeds of genus Castanea, $ Seeds of genus Corylus, ¥ Seeds of genus Carya (PDF) [file pone.0130942.s003.pdf]

Seeds	Mass (g)	Shell mass (g)	Hardness (kg)	Shell thickness (micro-meters)	Stratifi-cation period (days)	Energy (cal/g)	Protein (%)	Carbo-hydrates (%)	Lipid (%)	Tannins (%TAE)	Moisture (%)
F. grandifolia	0.26	0.13	18.76	292.89	90	7865.68	21.75	14.66	55.3	0.96	16.68
Castanea hybrid	1.77	0.6	12.11	186.59	60	5587.92	10.99	69.37	11.19	0.69	31.58
*C. dentata**	2.02	0.74	11.77	258.13	60	4253.57	13.16	65.39	12.53	0	24.52
*C. mollissima**	5.05	1.31	9.92	203.64	60	3653.47	6.3	81.71	4.38	0.36	33.72
N. densiflorus	2.75	1.88	22.3	1720.56	6	4999.5	5.31	65.5	23.05	11.54	30.35
Q. alba	2.52	1.48	14.9	575.42	0	4231.86	5.99	79.24	5.13	1.42	38.74
Q. bicolor	2.96	0.94	20.31	106.17	0	4162.15	6.55	76.23	7.1	2.72	42.32
Q. macrocarpa	1.74	0.53	19.94	209.3	45	4143.91	8.88	76	5.25	2.48	42.58
Q. michauxii	5.01	2.06	18.69	550.69	15	3854.02	4.4	86.4	4.6	2.33	42.69
Q. muehlenbergii	0.94	0.46	14.68	204.6	0	5651.24	7.87	75.61	7.28	5.14	40.18
Q. prinus	5.26	1.97	16.07	258.67	0	3602.57	5.87	82.07	2.41	3.87	40.02
Q. coccinea	2.53	1.18	19.46	427.24	45	5177.26	7.45	60.9	22.82	6.02	32.67
Q. palustris	1.73	0.59	13.78	378.82	120	5161.34	8.5	59.51	24.49	1.29	21.77
Q. rubra	6	1.97	14.87	468.91	50	5578.11	6.05	65.12	20.13	11.26	17.35
Q. velutina	0.72	0.35	15.26	302.22	45	5725.48	9.63	64.19	17	4.68	12.86
C. americana$	1.08	0.86	25.15	1170.27	90	8497.42	20.9	10.69	60.26	1	15.97
C. cordiformis¥	2.98	1.84	19.83	1209.85	90	5562.77	8.22	26.05	59.42	4.62	18.6
C. glabra¥	5.24	3.62	49.38	1838.89	105	6514.72	10.6	18.95	64.69	4.12	12.28
C. ovata¥	3.43	2.51	50.09	1783.96	105	8955.07	10.86	10.84	72.45	1.47	18.18
C. tomentosa¥	7.82	7.36	123.18	3108.67	120	8481.48	17.63	10.39	65.43	0	14.53
J. cinerea	14.41	13.71	211.83	1988.67	105	7764.48	29.96	4.3	58.08	0.39	14.66
J. nigra	19.56	18.45	148.64	2929.95	105	7200.18	27.92	8.22	56.34	0	14.63
J. regia	9.68	6.59	60.9	2178.64	93	8268.88	21.35	11.41	60.78	0.51	10.69
