# Supplementary material for: Segregating the Effects of Seed Traits and Common Ancestry of Hardwood Trees on Eastern Gray Squirrel Foraging Decisions
Source: PLoS One. 2015 Jun 25;10(6):e0130942. doi: 10.1371/journal.pone.0130942 (PMC4482146; doi:10.1371/journal.pone.0130942)
Supplement: S3 Table — Spearman rank correlation coefficients are in the upper diagonal of matrix and accompanying p-values in the lower diagonal. Boldface values correspond to significant correlations at an alpha of 0.05. In the table, kern = kernel mass (g), shell = shell mass (g), tannins = percentage TAE, energy = calories/g, hard = hardness (kg), thick = thickness (micrometers), interaction = hardness * thickness, dormancy = average cold stratification days, protein = percentage proteins in kernel, carb = percentage carbohydrate in kernel, lipid = percentage lipid in kernel, cons. time = consumption time (mins), cons. dist = distance to consume a seed (m), cache. time = time to cache (mins), dist. cache = distance to cache a seed (m). (PDF) [file pone.0130942.s004.pdf]

	kern	shell	tannins	energy	hard	thick	interaction	dormancy	protein	carb	lipid	cons. time	cons. dist.	cache time	dist. cache
kern		**0.611**	-0.067	-0.388	0.103	0.170	0.170	-0.093	-0.296	0.229	-0.253	**0.685**	**0.596**	**0.570**	**0.485**
shell	**0.002**		-0.207	0.306	**0.647**	**0.838**	**0.782**	**0.461**	0.172	**-0.474**	**0.503**	**0.855**	**0.578**	**0.703**	**0.840**
tannins	0.760	0.344		-0.249	-0.136	-0.173	-0.144	**-0.555**	**-0.657**	0.341	-0.183	-0.313	-0.046	-0.194	-0.255
energy	0.067	0.155	0.251		**0.574**	**0.594**	**0.548**	**0.663**	**0.753**	**-0.886**	**0.866**	0.114	-0.154	0.160	0.406
hard	0.641	**0.001**	0.538	**0.004**		**0.766**	**0.802**	**0.437**	**0.477**	**-0.690**	**0.672**	**0.545**	0.257	0.403	**0.570**
thick	0.438	**<0.001**	0.430	**0.003**	**<0.001**		**0.964**	**0.591**	0.388	**-0.729**	**0.739**	**0.551**	0.292	**0.502**	**0.679**
interaction	0.438	**<0.001**	0.511	**0.007**	**<0.001**	**<0.001**		**0.531**	**0.419**	**-0.712**	**0.670**	**0.524**	0.353	**0.433**	**0.618**
dormancy	0.672	**0.027**	**0.006**	**0.001**	**0.037**	**0.003**	**0.009**		**0.728**	**-0.822**	**0.801**	0.386	0.038	0.264	**0.542**
protein	0.170	0.433	**0.001**	**<0.001**	**0.021**	0.067	**0.047**	**<0.001**		**-0.800**	**0.629**	0.201	0.002	0.106	0.328
carb	0.293	**0.022**	0.111	**<0.001**	**<0.001**	**<0.001**	**<0.001**	**<0.001**	**<0.001**		**-0.920**	-0.296	0.010	-0.278	**-0.543**
lipid	0.244	**0.014**	0.403	**<0.001**	**<0.001**	**<0.001**	**<0.001**	**<0.001**	**0.001**	**<0.001**		0.308	0.005	0.215	**0.547**
cons. time	**0.000**	**<0.001**	0.146	0.606	**0.007**	**0.006**	**0.010**	0.069	0.359	0.170	0.152		**0.674**	**0.780**	**0.839**
cons. dist.	**0.003**	**0.004**	0.835	0.484	0.236	0.176	0.098	0.863	0.993	0.964	0.980	**<0.001**		0.339	**0.512**
cache time	**0.006**	**<0.001**	0.387	0.477	0.063	**0.017**	**0.044**	0.236	0.640	0.210	0.336	**<0.001**	0.123		**0.805**
dist. cache	**0.022**	**<0.001**	0.252	0.061	**0.006**	**0.001**	**0.002**	**0.009**	0.136	**0.009**	**0.008**	**<0.001**	**0.015**	**<0.001**	
