# Supplementary material for: Segregating the Effects of Seed Traits and Common Ancestry of Hardwood Trees on Eastern Gray Squirrel Foraging Decisions
Source: PLoS One. 2015 Jun 25;10(6):e0130942. doi: 10.1371/journal.pone.0130942 (PMC4482146; doi:10.1371/journal.pone.0130942)
Supplement: S4 Table — Traits include kernel mass (log), shell mass (log), tannin concentration (log), energetic or caloric concentration, hardness (log), shell thickness (log), interaction of hardness and shell thickness (log), dormancy period (given by number of cold stratification days), protein, carbohydrate and lipid concentration for each axis. Boldface values represent loadings that fall outside of 75th percentile cutoff values and are variables that are weighted heavily by the corresponding axis. (PDF) [file pone.0130942.s005.pdf]

| Seed traits | pPCA loadings | | |
| --- | --- | --- | --- |
| | pPC1 | pPC2 | pPC3 |
| Kernel | -0.030 | 0.234 | **0.659** |
| Shell | -0.292 | 0.114 | **0.392** |
| Tannins | 0.156 | **-0.667** | 0.093 |
| Energy | -0.320 | -0.121 | **-0.439** |
| Hardness | -0.318 | 0.006 | -0.309 |
| Shell thickness | **-0.341** | -0.158 | 0.242 |
| Interaction of hardness and thickness | -0.324 | -0.012 | 0.084 |
| Dormancy period | -0.321 | **-0.305** | 0.081 |
| Protein | -0.280 | **0.548** | -0.188 |
| Carbohydrate | **0.376** | 0.051 | -0.005 |
| Lipid | **-0.377** | -0.229 | 0.089 |
| $75^{th}$ percentile of loadings (absolute value) | 0.332 | 0.270 | 0.350 |
