# Supplementary material for: Segregating the Effects of Seed Traits and Common Ancestry of Hardwood Trees on Eastern Gray Squirrel Foraging Decisions
Source: PLoS One. 2015 Jun 25;10(6):e0130942. doi: 10.1371/journal.pone.0130942 (PMC4482146; doi:10.1371/journal.pone.0130942)
Supplement: S5 Table — * The p-value is given as proportion of null distribution (null distribution estimated by randomizing trait values across tips of the phylogeny) greater than estimated Moran’s I value. Boldface p-values are significant at alpha of 0.05. (PDF) [file pone.0130942.s006.pdf]

**Seed trait**	**Moran's I**	**p(>=I)***
Kernel mass (g)	-0.101	0.632
Shell mass (g)	0.469	**0.004**
Tannins (% TAE)	0.095	0.114
Energy (cal per dry gram)	0.540	**0.002**
Hardness (kg)	0.378	**0.006**
Shell thickness (micrometers)	0.652	**0.002**
Interaction of hardness and thickness	0.346	**0.002**
Dormancy period (days)	0.504	**0.002**
Protein (%)	0.556	**0.002**
Carbohydrate (%)	0.811	**0.002**
Lipid (%)	0.842	**0.002**
