# Supplementary material for: Segregating the Effects of Seed Traits and Common Ancestry of Hardwood Trees on Eastern Gray Squirrel Foraging Decisions
Source: PLoS One. 2015 Jun 25;10(6):e0130942. doi: 10.1371/journal.pone.0130942 (PMC4482146; doi:10.1371/journal.pone.0130942)
Supplement: S6 Table — * Sequences generated for this study. (PDF) [file pone.0130942.s007.pdf]

	ribulose-bisphosphate carboxylase (rbcL)	maturase K (matK)	internal transcribed spacer 2 (ITS 2)
Carya cordiformis	HQ590020	HQ593223	AF303820
Carya glabra	AF119186	**KR062084***	EU646156
Carya ovata	Li et al 2004	U92850	AF174620
Carya tomentosa	**KR062074***	AF118039	EU646134
Castanea dentata	KF613012	**KR062085***	**KR062093***
Castanea dentata and Castanea mollisima hybrid	**KR062075***	**KR062086***	NA
Castanea mollissima	HQ336406	EF057124	AY040396
Corylus americana	**KR062076***	AY212001	AF297349
Fagus grandifolia	HQ590099	HQ593295	AY232920
Juglans cinerea	HQ590142	AF118029	AF179572
Juglans nigra	HQ590143	U92851	AF338492
Juglans regia	HE963521	AF118038	HM049904

Notholithocarpus densiflorus	**KR062077***	FJ185047	AF389086
Quercus alba	EU676966	EU749360	AF098419
Quercus bicolor	**KR062078***	**KR062087***	**KR062094***
Quercus coccinea	**KR062079***	**KR062088***	**KR062095***
Quercus macrocarpa	HQ590229	HQ593405	HE611293
Quercus michauxii	**KR062080***	**KR062089***	EU646138
Quercus muehlenbergii	**KR062081***	**KR062090***	**KR062096***
Quercus palustris	AB125023	AB125040	AF098417
Quercus prinus (Q. montana)	**KR062082***	**KR062091***	AY040484
Quercus rubra	HQ590230	HQ593406	AF098418
Quercus velutina	**KR062083***	**KR062092***	EU646142
Rubus occidentalis	HQ590247	HQ593420	AF055758
